# Stereotactic body radiotherapy to defer systemic therapy in patients with oligorecurrent disease

**DOI:** 10.1016/j.ctro.2022.08.008

**Published:** 2022-08-19

**Authors:** Jonas Willmann, Eugenia Vlaskou Badra, Selma Adilovic, Sebastian M. Christ, Maiwand Ahmadsei, Michael Mayinger, Matthias Guckenberger, Nicolaus Andratschke

**Affiliations:** Department of Radiation Oncology, University Hospital Zurich, University of Zurich, Rämistrasse 100, 8091 Zurich, Switzerland

**Keywords:** Systemic Therapy-Free Interval, Oligorecurrent Disease, Stereotactic Body Radiotherapy, Oligometastatic Disease

## Abstract

**Background:**

Patients who develop oligorecurrent disease may be treated with metastasis-directed stereotactic body radiotherapy (SBRT) to defer the start of systemic therapy and delay its potential side effects. We report oncological outcomes and patterns of failure in patients with oligorecurrent disease treated with SBRT and determine which factors impact the interval to initiation of systemic therapy.

**Material/Methods:**

This retrospective study included patients with oligorecurrent disease (≤5 lesions) from any solid organ malignancy, treated with SBRT to all metastases and no systemic therapy for a minimum one month after SBRT between 01/2014 and 12/2019. The Kaplan-Meier method was used to analyze overall survival (OS) and progression-free survival (PFS), and the cumulative incidence of initiation of systemic therapy was analyzed assuming death without systemic therapy as a competing risk. Univariable and multivariable analyses are used to assess predictors of the systemic therapy-free interval.

**Results:**

Among 545 patients treated with SBRT for oligometastatic disease, 142 patients were treated with SBRT only for oligorecurrent disease. The most common primary tumors were lung and gastrointestinal cancer in 47 (33.1 %) and 28 (19.7 %) patients, respectively. After a median follow-up of 25 months, the median PFS and OS was 6.1 months and 48.9 months, respectively. Distant metastases were the most common first failure, and oligometastatic distant failure occured in 86 patients (60.6 %). New metastases were treated with repeat SBRT in 48 patients (33.8 %). The 1- and 2-year cumulative incidence of initiation of systemic therapy was 24.6 % and 36.8 %, respectively. In multivariable analysis, the number of previous lines of systemic therapy and the cumulative volume of metastases were significantly associated with the interval to initiation of systemic therapy.

**Conclusion:**

Selected patients with oligorecurrence achieved favorable OS and low cumulative incidence of initiation of systemic therapy. Prospective studies are warranted to determine how the deferral of systemic therapy impacts OS compared with immediate systemic therapy in combination with SBRT.

## Highlights

• SBRT may be used to defer systemic therapy in patients with oligorecurrence.
• Low rates of systemic therapy after SBRT for all oligorecurrent lesions were observed.
• Patients had favorable OS and few treatment-related toxicities.
• New metastases were treated with repeat SBRT in 33.8% of patients.
• Fewer lines of systemic therapy and a low baseline tumor volume were associated with longer systemic therapy-free interval.

## Background

Oligometastatic disease (OMD) refers to a state of limited metastatic spread, in which cancer patients might achieve long term survival or even cure through metastasis-directed local ablative therapies (LAT), such as stereotactic body radiotherapy (SBRT) [9]. LATs are commonly used in combination with systemic therapy agents, such as chemotherapy, targeted therapy, immunotherapy or hormonal therapy. A progression-free survival benefit from adding LATs to standard of care systemic therapies has been observed in randomized phase 2 trials [7], [10], [21], and phase 3 trials are currently underway to assess if LAT confers an overall survival benefit for OMD patients [15].

In other instances, LATs may be used to delay or defer systemic therapy in OMD patients, which has been endorsed as an endpoint in a consensus document by the European Society for Radiotherapy and Oncology (ESTRO) and American Society for Radiation Oncology (ASTRO) [14]. Systemic therapy-free survival might be particularly relevant for patients with oligorecurrent disease, i.e., those developing OMD while not on active systemic therapy [8]. As SBRT for treating oligometastases is usually well tolerated [13], this approach might yield favorable quality of life for patients, as the toxicities of systemic therapies are avoided, at least until patients develop widespread failure that can no longer be treated with SBRT or other LATs to all lesions.

Notable examples of successful application of LATs to defer systemic therapy come from the prospective trials by Ost [19] and Tang [23]: In the STOMP randomized phase 2 trial, Ost and colleagues used metastasis-directed SBRT or surgery in patients with metachronous oligorecurrent prostate cancer to nearly double androgen deprivation therapy (ADT)-free survival to 21 months compared with 13 months in patients undergoing surveillance. The single-arm phase 2 trial by Tang et al. used SBRT to defer systemic therapy in patients with oligorecurrent renal cell cancer. Importantly, repeat SBRT was allowed in case of repeat oligorecurrence. In the initial analysis of the trial, median progression-free survival was 22.7 months, no patients had died, and median systemic therapy-free survival was not reached, indicating that deferring systemic therapy by repeat SBRT is indeed feasible and well tolerated.

In the present study, we analyzed a cohort of patients with oligorecurrent disease from different primary tumors who were treated with SBRT without systemic therapy at our institution. We describe patient and treatment characteristics, oncological outcomes and patterns of failure, and determine which clinical variables impact the interval to initiation of systemic therapy after SBRT.

## Materials and methods

### Study design and patient selection

This retrospective single-center study assessed consecutive oligometastatic patients treated with metastasis-directed SBRT between 01/2014 and 12/2019 at the University Hospital Zurich. Inclusion criteria were oligorecurrent disease, i.e., development of new or progressive oligometastases in a systemic therapy-free interval, treatment of all lesions with SBRT and no systemic therapy-one month before diagnosis of oligorecurrent disease and minimum one month after SBRT. Further inclusion criteria were a maximum of 5 extracranial metastatic lesions and at least 18 years of age. There were no restrictions regarding primary tumor entities. Distant lymph node metastases were counted as distinct lesions. All regional lymph node metastases, if present, were counted as a single lesion. OMD states were determined according to the ESTRO and European Organisation for Research and Treatment of Cancer (EORTC) classification of OMD [8]. In brief, all patients had oligorecurrence, defined as development of OMD during a systemic therapy-free interval. Patients were further classified as metachronous oligorecurrence, i.e., the first time diagnosis of OMD (more than 6 months after initial diagnosis non non-metastatic cancer), and repeat oligorecurrence, after a previous history of OMD, and and induced oligorecurrence, after a history of polymetastatic disease.

This study followed the STROBE guideline for reporting of cohort studies and was approved by the institutional ethics board as well as the state ethics committee (BASEC ID 2018-01794).

### Treatment and follow-up

For the purpose of this study, SBRT was defined as the application of conformal treatment planning, image-guidance and stereotactic patient setup, using hypofractionated treatment application and inhomogeneous dose prescription. Non-ablative, palliative treatment regimens, e.g. 8 Gy in a single fraction or 5 × 4 Gy (homogeneously prescribed), were excluded. Using an alpha/beta ratio of 10, the biologically effective dose (BED10) was determined. Patients were followed up after SBRT with regular imaging and physical examination according to institutional guidelines. Generally, OMD patients are followed up every-three months for the first year after radiotherapy and every-six months thereafter or until progression, with clinical assessment and the imaging modality deemed appropriate by the treating primary oncologist and depending on the primary tumor location and histology, preferably FDG-PET/CT scan. Patients with oligometastatic prostate cancer receive Prostate-Specific Antigen (PSA) tests every-three months and PSMA-PET/CT or /MRI scans in case of biochemical recurrence.

### Statistical analysis

For descriptive statistics, median and range were used to describe continuous patient data variables and absolute counts and percentages for categorical patient data. Overall survival (OS) and progression-free survival (PFS) were calculated from the end of SBRT. Time-to-event curves were generated using the Kaplan-Meier method. The cumulative incidence of initiation of systemic therapy was analyzed assuming death without initiation of systemic therapy as a competing risk. The estimated variance of the cumulative incidence estimates were estimates of the asymptotic variance of Aalen [1]. For patients that died without having commenced systemic therapy, the reasons were determined.

Univariate and multivariate Cox proportional hazards models were applied to evaluate the impact on the systemic therapy-free interval after SBRT of ECOG performance status (0 vs 1 vs 2), age (continuous), primary tumor entity (lung cancer vs gastrointestinal cancer vs prostate cancer vs other), OMD state (metachronous oligorecurrence vs repeat oligorecurrence vs induced oligorecurrence), previous systemic treatment lines (0–1 vs 2 or more), involved organs (single vs multiple), metastatic lesions (single vs multiple), time since primary diagnosis (continuous) and the cumulative volume of metastases at baseline (cubic centimeters [cc], continuous). Multivariable analysis included primary tumor entity and OMD state, which we hypothesized to be putatively important, and factors that were significantly associated with the systemic therapy-free interval in univariable analysis. In general, the threshold for statistical significant differences was set at p ≤ 0.05. To account for multiple testing in the univariable analysis, Bonferroni correction was applied, resulting in a threshold of p ≤ 0.004 for statistical significance in the univariable analysis.

All statistical analyses were performed in R (R version 4.03.00; R Development Core Team), with the “survival”, “survminer”, “cmprsk”, “clinfun” and “finalfit” packages.

## Results

### Patient and treatment characteristics

We screened the records of 545 patients who were treated with SBRT for extracranial oligometastatic disease at our hospital between January 2014 and December 2019. In total, 142 patients were eligible due to oligorecurrent disease and were included in the study. Patient characteristics are outlined in Table 1. The median age was 68 years (interquartile range [IQR] 62–74), 31.7 % (n = 45) were female. The most common primary tumors were lung (n = 47, 33.1 %), non-colorectal gastrointestinal (n = 28, 19.7 %) and prostate cancers (n = 17, 12.0 %). The median time from primary diagnosis to presentation with OMD were 31 months. Seventy-six patients (53.5 %) presented with metachronous oligorecurrence, 54 (38.0 %) with repeat oligorecurrence and 12 (8.5 %) with induced oligorecurrence. The majority of patients were staged using PET-CT (n = 79, 55.6 %) or CT (n = 52, 36.7 %). Most patients had received one line of systemic therapy prior to the SBRT for oligorecurrence (50.7 %, n = 72), while 50 patients (35.2 %) had not received any prior systemic therapy. Sixteen patients (11.2 %) had two prior lines of systemic therapy and 4 patients (2.8 %) had three or more lines.

**Table 1:** Patient characteristics. Data are in n (%) or median (IQR). Abbreviations: ECOG PS: Eastern Cooperative Oncology Group performance status.

		Patients (n = 142)
Age (years)	Median (IQR)	68 (62–74)
Sex	Male	97 (68.3)
	Female	45 (31.7)
OMD state	Metachronous oligorecurrence	76 (53.5)
	Repeat oligorecurrence	54 (38.0)
	Induced oligorecurrence	12 (8.5)
Primary tumor	Lung	47 (33.1)
	Gastrointestinal (non-colorectal)	28 (19.7)
	Prostate	17 (12.0)
	Head and neck	13 (9.2)
	Colorectal	12 (8.5)
	Urogenital (non-prostate)	7 (4.9)
	Melanoma	3 (2.1)
	Breast	1 (0.7)
	Other	14 (9.9)
Number of metastatic lesions	1	95 (66.9)
	2	38 (26.8)
	3	6 (4.2)
	4	3 (2.1)
Number of involved organs	1	128 (90.1)
	2	14 (9.9)
Primary controlled	Yes	128 (90.1)
	No	14 (9.9)
ECOG PS	0	58 (40.8)
	1	42 (29.6)
	2	8 (5.6)
	Unknown	34 (23.9)
Number of systemic treatment lines	1	72 (50.7)
	0	50 (35.2)
	2 or more	20 (14.1)

A total of 192 lesions were treated in the 142 oligorecurrent patients; in all patients, all progressive or persistent metastases were treated. Radiotherapy characteristics per lesion are shown in Table 2. The median number of fractions was 5.0 (IQR 3–5) and the median dose per fraction was 8.0 (IQR 6–9.5), resulting in a median prescription BED10 of 68.4 Gy (59.5–84.4). The treated lesions had a median GTV volume of 3.9 cc (IQR 1.4–13.4). Lung, bone and liver were the most commonly treated organs, in 107 (54.9 %), 29 (14.9 %) and 19 (9.7 %) of cases, respectively.

**Table 2:** Treatment characteristics per metastatic lesion. Data are in n (%) or median (IQR). Abbreviations: GTV: gross tumor volume; BED10: prescription biologically effective dose, using an alpha/beta ratio of 10, cc: cubic centimeters.

		**Metastases (n = 195)**
*Number of fractions*	Median (IQR)	5.0 (3.0–5.0)
*Dose per fraction (Gy)*	Median (IQR)	8.0 (6.0–9.5)
*Total dose (Gy)*	Median (IQR)	37.5 (35.0–43.2)
*BED10 (Gy)*	Median (IQR)	68.4 (59.5–84.4)
*GTV volume (cc)*	Median (IQR)	3.9 (1.4–13.4)
*Location*	Lung	107 (54.9)
	Bone	29 (14.9)
	Liver	19 (9.7)
	Lymph nodes	14 (7.2)
	Pleura	11 (5.6)
	Adrenal gland	9 (4.6)
	Soft tissue	3 (1.5)
	Kidney	1 (0.5)
	Pancreas	1 (0.5)
	Pericardium	1 (0.5)

### Oncological outcomes and patterns of failure

**Fig. 1:**
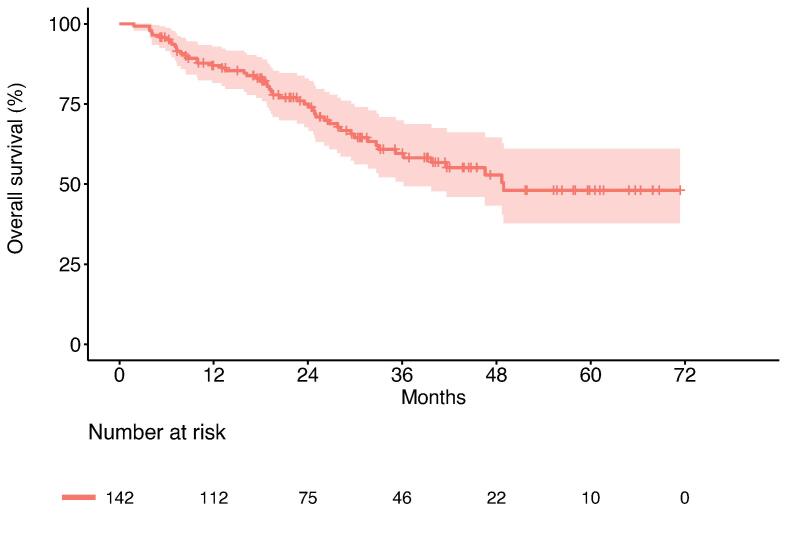
Kaplan-Meier plot for overall survival (OS). Pale area indicates 95% confidence interval.

**Fig. 2:**
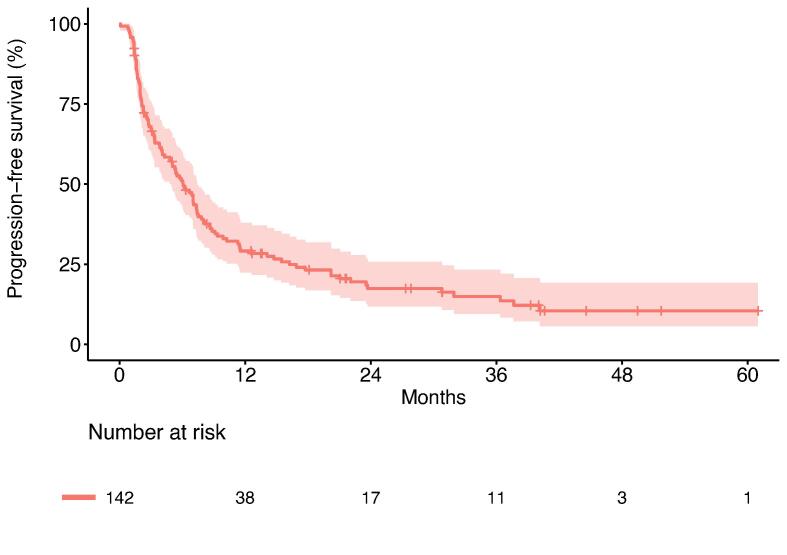
Kaplan-Meier plot for progression-free survival (PFS). Pale area indicates 95% confidence interval.

After a median follow-up of 25 months, 52 patients died and 115 had disease progression. The median OS was 48.9 months (95 % CI: 36.2 - not reached [NR]), with 1- and 2-year OS rates of 87.0 % (95 % CI: 81.5 % − 92.8 %) and 75.0 % (95 % CI: 67.7–83.0 %), respectively (Fig. 1). Patients had a median PFS of 6.1 months (95 % CI 5.0–7.4), the 1- and 2-year PFS rates were 29.1 % (95 % CI: 22.4 % − 37.9 %) and 17.4 % (95 % CI: 11.8 % − 25.7 %), respectively (Fig. 2). The most common pattern of first failure was distant progression (n = 80, 56.3 %). Other patterns of first failure were combined failure with distant and locoregional progression (n = 22, 15.5 %), or locoregional progression of the primary tumor alone in 9 patients (15.5 %). Only three patients (2.1 %) had local progression of the SBRT treated metastases as their first failure. The majority of patients (n = 86, 60.6 %) presented again with oligometastatic disease of a maximum of 5 new lesions at distant failure.

### Cumulative incidence of initiation of systemic therapy

**Fig. 3:**
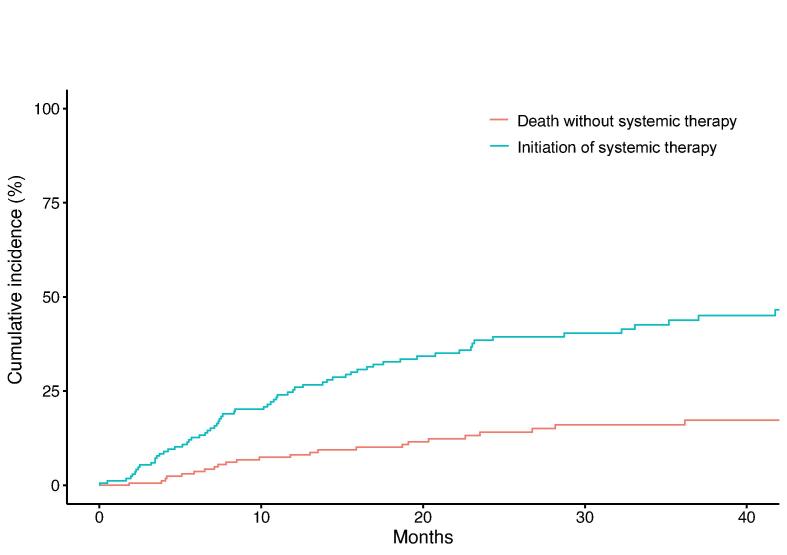
Cumulative incidence of systemic therapy initiation, accounting for the competing risk of death without initiation of systemic therapy.

Eventually, 54 patients (38.0 %) started a new line of systemic therapy while 24 (30.8 %) died without having commenced systemic therapy. The 1- and 2-year cumulative incidence of initiation of systemic therapy was 24.6 % and 36.8 % (estimated variance: 0.01 %, 0.02 %), respectively (Fig. 3). The cumulative incidence of death without initiation of systemic therapy at 1 and 2 years was 8.7 % and 15.8 % (estimated variance: 0.01 %, 0.01 %), respectively.

When systemic therapy was initiated, 30 patients (21.1 %) received chemotherapy alone or in combination with targeted therapy or immune-checkpoint inhibitors. Carboplatin was the most commonly applied chemotherapeutic agent (n = 15). Thirteen patients (9.2 %) were treated with immune-checkpoint inhibitors alone, 4 patients (2.8 %) with targeted therapy alone. The most commonly used immune-checkpoint inhibitors were Nivolumab (n = 7) and Pembrolizumab (n = 6). Hormonal therapy was started in 7 patients (5.0 %). New metastases were treated with repeat SBRT to all lesions in 48 patients (33.8 %). After the initial course of SBRT one (0.5 %) grade 3 potentially radiation-induced toxicity occurred.

Twenty-four (16.9 %) patients had died without having commenced a new line of systemic therapy. We compared their baseline characteristics to patients who eventually started systemic therapy, and those who were alive without systemic therapy at their last follow-up. Significantly fewer patients who died without systemic therapy had no previous lines of systemic treatment, otherwise patient characteristics were balanced (Appendix Table 1). Among patients that died without systemic therapy, 12 (50 %) did so because of exacerbated comorbidities. In 5 cases (20.8 %), patients declined further systemic therapy. In three cases (12.5 %), no further line of systemic therapy was deemed safe because of the patient's dismal performance status. For 4 patients (16.7 %), the reasons for not starting systemic therapy prior to death could not be determined from the patient records, as they were lost to follow-up. In the appendix, we also report systemic therapy-free survival - using both initiation of systemic therapy and death as an event (Fig. A1).

### Factors associated with the systemic therapy-free interval following SBRT

We analyzed the association of patient characteristics with the systemic therapy-free interval following SBRT to all metastases (Table 3). In exploratory univariable analysis, the number of previous lines of systemic therapy (two or more vs none or one) and the cumulative volume of metastases reached the Bonferroni-corrected threshold for statistical significance of 0.004. In multivariable analysis, the number of previous systemic therapy lines both variables remained strong predictors of the systemic therapy-free interval: higher cumulative volume of metastases at baseline (HR: 1.02 (1.01–1.03, p = 0.002) and two or more previous lines of systemic therapy (HR 2.17, 95 % CI 1.04–4.53, p = 0.039) were associated with significantly shorter systemic therapy-free interval. Repeat oligorecurrence was not significantly associated with the systemic therapy-free interval in multivariable analysis (HR 0.44, 95 % CI 0.17–1.16, p = 0.10), as was primary tumor entity.

**Table 3:** Univariable and multivariable analyses of parameters associated with STFS. Abbreviations: ECOG PS: Eastern Cooperative Oncology Group performance status; SD: standard deviation; cc: cubic centimeters.

			**Systemic therapy-free interval HR (95 %-CI, p-value)**	
		n (%)	*Univariable*	*Multivariable*
**Age (years)**	*Mean (SD)*	67.3 (10.7)	0.99 (0.97–1.01, p = 0.44)	
**ECOG PS**	*0*	58 (40.8)	*Reference*	
	*1*	42 (29.6)	1.13 (0.57–2.22, p = 0.73)	
	*2*	8 (5.6)	0.44 (0.06–3.24, p = 0.42)	
**Primary tumor**	*Lung*	47 (33.1)	*Reference*	*Reference*
	*Prostate*	17 (12.0)	0.93 (0.49–1.78, p = 0.83)	0.85 (0.43–1.68, p = 0.64)
	*Gastrointestinal (non-colorectal)*	28 (19.7)	0.82 (0.34–1.97, p = 0.66)	1.05 (0.42–2.59, p = 0.92)
	*Other*	50 (35.2)	0.96 (0.44–2.08, p = 0.92)	1.04 (0.46–2.32, p = 0.93)
**OMD state**	*Induced oligorecurrence*	12 (8.5)	*Reference*	*Reference*
	*Metachronous oligorecurrence*	76 (53.5)	0.39 (0.17–0.90, p = 0.03)	0.64 (0.24–1.67, p = 0.36)
	*Repeat oligorecurrence*	54 (38.0)	0.32 (0.13–0.77, p = 0.01)	0.44 (0.17–1.16, p = 0.10)
**Previous lines of systemic therapy**	*0*–*1*	122 (85.9)	*Reference*	*Reference*
	*2 or more*	20 (14.1)	2.72 (1.43–5.18, p = 0.002)	2.17 (1.04–4.53, p = 0.04)
**Involved organs**	*Single*	128 (90.1)	*Reference*	
	*Multiple*	14 (9.9)	2.55 (1.20–5.42, p = 0.015)	
**Number of metastatic lesions**	*Single*	95 (66.9)	*Reference*	
	*Multiple*	47 (33.1)	1.08 (0.62–1.91, p = 0.78)	
**Time since primary diagnosis (months)**	*Mean (SD)*	49.1 (49.2)	1.00 (0.99–1.01, p = 0.92)	
**Cumulative volume of metastases (cc)**	*Mean (SD)*	15.4 (20.4)	1.02 (1.01–1.03, p < 0.001)	1.02 (1.01–1.03, p = 0.002)

## Discussion

We report an analysis of highly selected patients with oligorecurrent metastatic disease from different primary tumors who were treated with metastasis-directed SBRT alone without immediate systemic therapy. Despite a relatively short median PFS of 6.1 months, the cumulative incidence of initiation of systemic therapy was considerably low. The predominant pattern of first failure after SBRT were new distant metastases, often repeat oligometastases. More than a third of patients were treated with repeat SBRT to new lesions, which might have facilitated a systemic therapy-free interval beyond disease progression in some cases. Even if only 36.8 % of patients had started systemic therapy after 2 years, we observed favorable survival rates in our cohort, with a median OS of 48.9 months. Baseline patient factors predicting longer systemic therapy-free interval were fewer previous lines of systemic therapy and a lower cumulative volume of metastases.

Systemic therapy is a mainstay in the treatment of metastatic cancer, thus its deferral might adversely affect clinical outcomes. However, delaying the initiation of systemic therapy might be valuable especially in patients with less effective and potentially more toxic further-line systemic therapy options. In the randomized TOAD trial, delayed compared with immediate ADT in prostate cancer patients with PSA-only relapse after definitive treatment, or de-novo non-curable disease, was associated with fewer early adverse hormone-treatment-related symptoms, and did not affect overall functioning or quality of life [5]. A retrospective observational study utilizing the National Cancer Data Base found that delayed targeted therapy was not associated with worse OS in patients with metastatic renal-cell carcinoma [26]. A retrospective single center analysis from MD Anderson Cancer Center found that in patients with asymptomatic advanced gastric cancer, delayed systemic therapy did not result in detrimental OS [6]. These studies indicate that in carefully selected patients with advanced cancer, outcomes might not be compromised by surveillance and delayed systemic therapy.

The impact of using LAT of metastatic lesion - potentially repeatedly - rather than surveillance to delay systemic therapy is not well studied. To the best of our knowledge, the STOMP trial [19] has been the only instance comparing LAT and surveillance in a prospective, randomized setting in patients with prostate cancer. Several smaller, retrospective series reported outcomes of oligorecurrent patients treated with SBRT without systemic therapy. In a retrospective series on SBRT for oligorecurrent prostate cancer, concomitant ADT with SBRT was associated with improved PFS and widespread failure-free survival, compared with delayed ADT [2]. Another retrospective series analyzed patients treated with repeat LAT, including SBRT, for pulmonary oligorecurrence from different primary tumors [16]. About half of the patients (44.1 %) had a repeat pulmonary oligorecurrence and received multiple courses of LAT. The 3-year STFS was longer in patients receiving multiple courses of SBRT than in the single course group (50.4 % vs 44.7 %), albeit these differences did not reach statistical significance. The 3-year OS rate was favorable in patients treated with single or repeat course SBRT (73.9 % vs 78.8 %), while PFS rates were not reported. Lazzari and colleagues reported outcomes of patients with oligometastatic ovarian cancer treated with metastasis-directed SBRT [12]. Among the subgroup of oligorecurrent patients the median STFS was 7.4 months; in 23 patients (28 %) systemic therapy was deferred by at least 1 year. Other oncological outcomes were not specifically reported with oligorecurrent patients, but in the entire cohort the actuarial 2-year PFS, and OS rates were 18 %, and 71 %, respectively. SBRT was well tolerated and no grade 3 or 4 acute or late events were observed.

A retrospective multicenter series by Kissel and colleagues reported on patients with oligometastatic lung cancer who were treated with SBRT with or without systemic therapy [11]. The median OS and distant progression-free survival were 28.2 months and 6.3 months, respectively. Patients treated with repeat LAT for repeat oligometastatic disease had a trend for better survival compared with single course of LAT patients. The mean systemic therapy-free interval in oligorecurrent patients was 11.2 months (range 1 to 40 months). No grade 3 or 4 toxicities were observed, but notably, one toxic treatment-related death occurred after SBRT of a sphenoid lesion. Taken together, these retrospective series corroborate our findings and indicate that SBRT for oligorecurrence is usually well tolerated and may yield long-term systemic therapy-free intervals and OS in selected patients. Post-progression treatments such as repeat SBRT in patients developing consecutive oligorecurrence may facilitate survival without systemic therapy beyond disease progression.

In other instances, metastases-directed SBRT has been used to prolong not the interval without systemic therapy, but allow for the continuation of systemic therapy beyond disease progression. Deek et al., retrospectively analyzed patients with oligoprogressive castration-resistant prostate cancer (CRPC), treated with metastases-directed SBRT [4]. The time to next systemic therapy was comparable in patients that continued their systemic therapy and received metastases-directed SBRT as compared to those with a change of systemic therapy alone. These results indicate that SBRT may be used to prolong the time patients can remain on their current systemic therapy. Compared with a change in systemic therapy alone, SBRT was associated with longer to time to PSA failure. In an interim analysis of the prospective randomized phase 2 trial CURB (to date only presented as an abstract), including patients with oligoprogressive non-small cell lung cancer (NSCLC) of breast cancer, metastasis-directed SBRT was associated with improved PFS compared with standard systemic treatment [24]. Interestingly, this effect was driven by the substantial response in NSCLC patients, while breast cancer patients derived no benefit. These results highlight that more work is needed to determine which patients and tumor types may benefit from metastasis-directed LATs.

If future trials would establish that delaying systemic therapy by metastasis-directed SBRT in selected patients with oligorecurrence were non-inferior to combined modality treatments regarding OS, the cost-effectiveness of such an approach should be analyzed. The cost-effectiveness of SBRT in addition to standard of care systemic therapy for patients with oligometastatic disease has been investigated in two studies based on the SABR COMET trial. The data by Mehrens et al. and Qu et al. suggest that adding metastasis-directed SBRT might be cost-effective [17], [22]. Another study analyzed the cost-effectiveness of using LAT to delay systemic therapy (ADT) in patients with oligorecurrent prostate cancer was cost-effective, based on the STOMP trial. Indeed, De Bleser et al. [3] found that LAT alone is potentially cost-effective compared with immediate ADT [3]. Importantly, the impact on clinical endpoints such as OS of definitive SBRT and delayed systemic therapy compared with immediate combined treatments needs to be determined before its cost-effectiveness can be investigated.

Our study is limited by its retrospective nature and small sample size. Patients are highly selected and the reasons for initial omission of systemic therapy cannot be determined. Notably, the indications for initiating systemic therapy were not standardized. When definitive SBRT is tested in a prospective setting, rigorous criteria for starting systemic therapy should be prespecified, e.g. a certain number or velocity of new metastases. The generalizability of our results is also limited by the heterogeneity of our study population, particularly as the relatively small sample size hampers subgroup analysis of different primary tumors and OMD states: patients with repeat oligorecurrence at baseline might have a favorable prognosis [25] and thus benefit from repeat SBRT, albeit their systemic therapy-free interval was not significantly different from induced oligorecurrence patients in the present study (p = 0.10). The lack of a control group in our study also prohibits assumptions on whether the delay of systemic therapy might adversely affect OS, which should at best be addressed in a prospective, randomized setting. Our results may be hypothesis generating for future prospective clinical trials, which should compare relevant oncological outcomes such as OS and quality of life in patients treated with SBRT and delayed systemic therapy compared with those receiving a combined treatment of SBRT and immediate systemic therapy.

## Conclusion

Highly selected patients had relatively low rates of initiation of systemic therapy following definitive SBRT for all oligorecurrent lesions, and achieved favorable OS and few treatment-related toxicities. Fewer previous lines of systemic therapy and a lower cumulative volume of metastases at baseline were associated with longer systemic therapy-free intervals. The role of repeat SBRT for new metastases to further delay systemic therapy warrants further investigation. Prospective studies are needed to determine whether systemic therapy might be deferred after SBRT to all metastases in selected patients with oligorecurrence without compromising OS or quality of life.

## Declaration of Competing Interest

The authors declare the following financial interests/personal relationships which may be considered as potential competing interests:

Dr. Andratschke reports personal fees from AstraZeneca, personal fees from Debiopharm, grants, personal fees and non-financial support from ViewRay, grants and personal fees from Brainlab, outside the submitted work

The remaining authors declare that they have no known competing financial interests or personal relationships that could have appeared to influence the work reported in this paper.
