# Supplementary material for: Stereotactic body radiotherapy to defer systemic therapy in patients with oligorecurrent disease
Source: Clin Transl Radiat Oncol. 2022 Aug 19;37:12–8. doi: 10.1016/j.ctro.2022.08.008 (PMC9421088; doi:10.1016/j.ctro.2022.08.008)

Supplementary material

Table A1

Patient characteristics of patients that were alive without systemic therapy at last follow-up, had systemic therapy initiated or had died without having started systemic therapy. Data are in n (%) or median (IQR). Abbreviations: ECOG PS: Eastern Cooperative Oncology Group performance status.

	´	Alive without systemic therapy	Initiation of systemic therapy	Death without systemic therapy	p-value
		64 (45.1)	54 (38.0)	24 (16.9)	
Age (years)	Median (IQR)	66.9 (12.4)	66.5 (9.4)	70.4 (8.1)	0.314
Sex	Male	41 (64.1)	41 (75.9)	15 (62.5)	0.308
	Female	23 (35.9)	13 (24.1)	9 (37.5)	
OMD state	Metachronous oligorecurrence	32 (50.0)	30 (55.6)	14 (58.3)	0.366
	Repeat oligorecurrence	29 (45.3)	17 (31.5)	8 (33.3)	
	Induced oligorecurrence	3 (4.7)	7 (13.0)	2 (8.3)	
Primary tumor	Lung	19 (29.7)	18 (33.3)	10 (41.7)	0.132
	Gastrointestinal	10 (15.6)	10 (18.5)	8 (33.3)	
	Prostate	10 (15.6)	7 (13.0)	0 (0.0)	
	Head and neck	8 (12.5)	4 (7.4)	2 (8.3)	
	Colorectal	4 (6.2)	7 (13.0)	2 (8.3)	
	Urogenital (non-prostate)	7 (10.9)	4 (7.4)	1 (4.2)	
	Melanoma	6 (9.4)	0 (0.0)	1 (4.2)	
	Breast	0 (0.0)	3 (5.6)	0 (0.0)	
	Other	0 (0.0)	1 (1.9)	0 (0.0)	
Number of metastatic lesions	1	43 (67.2)	36 (66.7)	16 (66.7)	0.600
	2	18 (28.1)	15 (27.8)	5 (20.8)	
	3	1 (1.6)	3 (5.6)	2 (8.3)	

	4	2 (3.1)	0 (0.0)	1 (4.2)	
Number of involved organs	1	60 (93.8)	46 (85.2)	22 (91.7)	0.287
	2	4 (6.2)	8 (14.8)	2 (8.3)	
Primary controlled	Yes	59 (92.2)	47 (87.0)	22 (91.7)	0.622
	No	5 (7.8)	7 (13.0)	2 (8.3)	
ECOG PS	0	28 (43.8)	21 (38.9)	9 (37.5)	0.269
	1	18 (28.1)	14 (25.9)	10 (41.7)	
	2	13 (20.3)	18 (33.3)	3 (12.5)	
	Unknown	5 (7.8)	1 (1.9)	2 (8.3)	
Number of systemic treatment lines	1	31 (48.4)	26 (48.1)	15 (62.5)	0.009
	0	30 (46.9)	16 (29.6)	4 (16.7)	
	2 or more	3 (4.7)	12 (22.2)	5 (20.8)	

Figure A1

Kaplan-Meier plot for systemic therapy-free survival (STFS). Pale area indicates 95% confidence interval. Median STFS was 23.0 months (95% CI 18.6-36.1), and STFS rates at 1 and 2 years were 66.7% (95% CI 59.2-75.1) and 47.3% (95% CI 39.2-57.1), respectively.

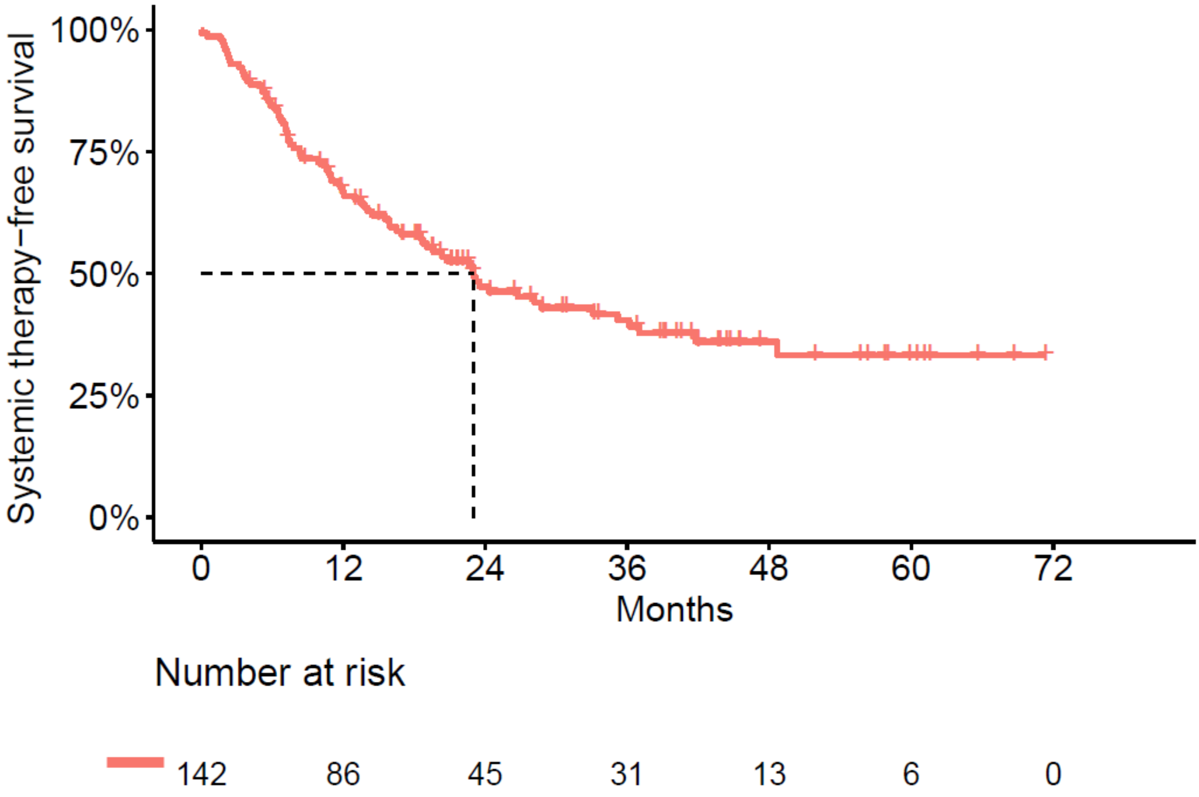

Supplement: Supplementary data 1 [file mmc1.pdf]
